# Exploring novel natural compound-based therapies for Duchenne muscular dystrophy management: insights from network pharmacology, QSAR modeling, molecular dynamics, and free energy calculations

**DOI:** 10.3389/fphar.2024.1395014

**Published:** 2024-10-02

**Authors:** Mohd Saeed, Ashanul Haque, Ambreen Shoaib, Syed Mohd Danish Rizvi

**Affiliations:** ^1^ Department of Biology, College of Sciences, University of Ha’il, Ha’il, Saudi Arabia; ^2^ Department of Chemistry, College of Sciences, University of Ha’il, Ha’il, Saudi Arabia; ^3^ Department of Clinical Pharmacy, College of Pharmacy, Jazan University, Jazan, Saudi Arabia; ^4^ Department of Pharmaceutics, College of Pharmacy, University of Ha’il, Ha’il, Saudi Arabia

**Keywords:** Duchenne muscular dystrophy, network pharmacology, computational drug discovery, pharmacophore modeling, molecular dynamics simulation, QSAR

## Abstract

Muscular dystrophies encompass a heterogeneous group of rare neuromuscular diseases characterized by progressive muscle degeneration and weakness. Among these, Duchenne muscular dystrophy (DMD) stands out as one of the most severe forms. The present study employs an integrative approach combining network pharmacology, quantitative structure-activity relationship (QSAR) modeling, molecular dynamics (MD) simulations, and free energy calculations to identify potential therapeutic targets and natural compounds for DMD. Upon analyzing the GSE38417 dataset, it was found that individuals with DMD exhibited 290 upregulated differentially expressed genes (DEGs) compared to healthy controls. By utilizing gene ontology (GO) and protein-protein interaction (PPI) network analysis, this study provides insights into the functional roles of the identified DEGs, identifying ten hub genes that play a critical role in the pathology of DMD. These key genes include DMD, TTN, PLEC, DTNA, PKP2, SLC24A, FBXO32, SNTA1, SMAD3, and NOS1. Furthermore, through the use of ligand-based pharmacophore modeling and virtual screening, three natural compounds were identified as potential inhibitors. Among these, compounds 3874518 and 12314417 have demonstrated significant promise as an inhibitor of the SMAD3 protein, a crucial factor in the fibrotic and inflammatory mechanisms associated with DMD. The therapeutic potential of the compounds was further supported by molecular dynamics simulation and Molecular Mechanics/Generalized Born Surface Area (MM/GBSA) analysis. These findings suggest that the compounds are viable candidates for experimental validation against DMD.

## 1 Introduction

Muscular dystrophies (MD) refer to a variety of inherited conditions that cause progressive weakening and degeneration of skeletal muscles. This group of disorders is known for its genetic and clinical diversity, with symptoms appearing from birth to late adulthood depending on the specific type. The most diverse forms of MD include limb-girdle, facioscapulohumeral, oculopharyngeal, emery-Dreifuss, distal, and Duchenne and Becker muscular dystrophies (Angelini, 2010). MD encompass a range of genetic disorders that can be X-linked, autosomal recessive, or autosomal dominant. These conditions manifest as muscular discomfort, weakness, and degeneration. Mutations in proteins within the sarcomere, nucleus, basement, or outer membrane of muscle cells and nonstructural enzymatic proteins are implicated in the pathogenesis of MD (Amato and Griggs, 2011). Different genetic deletions or mutations can lead to enzymatic or metabolic disorders, resulting in a variety of MDs (Gao and McNally, 2015; Allen et al., 2016). Variation in the X-lined gene DMD, where dystrophin is mutated frequently, causes Duchenne muscular dystrophy (DMD), which is the most severe form (Brinkmeyer-Langford and Kornegay, 2013). This condition affects around 1 in every 3,500 to 5,000 male infants born globally (Hoffman et al., 1987). Downregulation of protein expression during DMD or BMD is an outcome of mutations associated with Dytrophin gene (Le Rumeur, 2015). Limb-girdle muscular dystrophy (LGMD) is a progressive muscle weakness affecting the pelvic and shoulder girdles, primarily caused by mutations in proteins like myotilin, lamin, caveolin-3, calpain-3, dysferlin, γ-sarcoglycan, TCAP, TRIM32, FKRP, and titin. These mutations cause various subtypes of LGMD and are associated with other forms of muscular dystrophies, including distal and congenital dystrophies (Lovering et al., 2005).

Genetic profiling has revealed significant changes in gene expression in DMD. These changes offer insights into the disease's molecular basis and highlight potential therapeutic targets. Altered gene expression contributes to characteristic features of the disease. These altered gene expression leads to inflammation, fibrosis, and muscle degeneration. Studies have identified multiple genes associated with these biological processes, suggesting potential therapeutic interventions. Wang et al. (2013) found several hub genes associated with DMD, including C3AR1, TLR7, IRF8, and CD33, which are linked to immune and inflammation responses. Wang et al. (2021) identified proteins acting as hubs for DMD and BMD, finding 1,281 genes overexpressed and 189 downregulated. The informational evolution underscores the complexity of DMD and the need for ongoing research to understand its molecular intricacies.

Based on the immunomodulators of DMD pathology, various drug targets were used in phase I and preclinical trials (Malik et al., 2012; Tripodi et al., 2021). Inhibiting the SMAD3 signaling pathway to help reduce inflammation and fibrosis in DMD patients. SMAD3 is a protein with two domains, MH1 and MH2, crucial for TGF-β-induced transcriptional activation. The MH2 domain interacts with transcriptional cofactors and the type I TGF-β receptor. It binds to various proteins without a common sequence motif, making it essential for activating TGF-β signals, as demonstrated by studies the domain of SMAD3 in transforming growth factor-β signaling (Imoto et al., 2005a).

Halofuginone was tested in the mouse model to inhibit SMAD3 signaling, and it was observed that the inhibition of SMAD3 reduced fibrosis and improved muscle function (Tripodi et al., 2021). Osseni et al. (2022) found that tubastatin A can inhibit HDAC6, thereby enabling the acetylation of SMAD3. It also prevents nuclear translocation and Smad2/3 phosphorylation, reducing muscle atrophy and fibrosis by downregulating TGF-β signaling through Smad3 acetylation. Overall, both studies showed that inhibiting SMAD3 restricts the progression of fibrosis improves muscle movement.

This study aims to focus on the SMAD3 gene, which is a key mediator in the TGF-β signaling pathway in DMD. The gene expression data from the GEO database was used to identify differentially expressed genes and to construct a Protein-Protein Interaction network. Molecular docking was used to screen 2,569 natural compounds against SMAD3’s MH2 domain, selecting three compounds with strong binding affinities. The study validated these compounds’ potential as therapeutic agents through molecular dynamics simulations, principal component analysis (PCA), free energy landscape analysis (FEL analysis), and MM/GBSA calculations. This approach addresses a gap in current management strategies for DMD and sets the stage for developing more targeted and effective therapeutic options. The study’s integrative approach combines high-throughput gene expression analysis, network-based bioinformatics, and advanced computational docking and simulation methods.

## 2 Methodology

### 2.1 Data collection, preprocessing and differential gene expression analysis

In order to find the gene expression data, a search was performed in the Gene Expression Omnibus (GEO) database using the keyword “muscular dystrophy” (Clough and Barrett, 2016). The data was filtered specifically for the species “*Homo sapiens*.” The microarray dataset with the accession number GSE38417 was selected for further examination based on the data processing. The selected dataset contains the microarray datasets of healthy (control) and active MD patients. The microarray gene expression data from the selected groups were analysed using the GEO2R tool to determine the DEGs (Rezaei and Jabbari, 2022). The GEO2R software was used to obtain the *p*-value and false discovery rate (FDR) using the *T*-test and the Benjamini and Hochberg technique (Aubert et al., 2004). DEGs were selected by using a cut-off value of an absolute value of the log(fold change) | > 2 and a significance threshold of *p* < 0.05. DEGs were classified as upregulated or downregulated according to logFC ≥ 2 and logFC ≤ −2, respectively.

### 2.2 Protein-protein interaction network analysis, gene ontology and pathway analysis

This study integrated GEO for expression data, STRING for building interaction networks, and Cytoscape for network visualization (Menche et al., 2015). In order to examine the protein-protein interaction (PPI) network of DEGs, the search Tool for the Retrieval of Interacting Genes’ (STRING, version 12.0) (Szklarczyk et al., 2021) was utilised. The PPI network was visualised using the web-based software Cystoscope. The next step was to locate the hub genes in Cytoscape (Shannon et al., 2003) by using the MCC method and the CytoHubba plugin (Chin et al., 2014). Further, gene ontology was employed to evaluate the roles of the genes.

Here, the GO approach was used to analyze the molecular function of the gene. To perform GO analysis, the g:Profiler server (Reimand et al., 2007) was used, and the GOST tool was used to define the advanced parameters. Over-representation analysis (ORA), also known as gene set enrichment analysis, functional enrichment analysis, and other similar analyses, are all services provided by the GOST programme (Raudvere et al., 2019). This analysis is carried out on a list of input genes. Annotated genes were the primary ones chosen in this case under the advanced option. After that, g:SCS was selected as a significance threshold and set the user threshold to 0.5. When determining the *p*-values, the default parameter was used as the starting point, and a false discovery rate (FDR) threshold of *p* < 0.05 was implemented in order to analyze the data. A statistically significant *p*-value of less than 0.05 was achieved by using the ShinyGO 0.80 programme to carry out the route analysis (Ge et al., 2020). The pathway database known as the Kyoto Encyclopaedia of Genes and Genomes (KEGG) was used by this algorithm in order to discover the genes that were implicated (Kanehisa and Goto, 2000). The KEGG pathway representation predominantly focuses the intricate relationship of gene products, primarily proteins, while additionally includes functional RNAs (Kanehisa and Goto, 2000). The FDR cut-off in the KEGG pathway has been set as 0.05. Consequently, relevant genes were acquired, and among them, additional selection was conducted based on the enrichment score (0.05).

### 2.3 Target preparation

The top hub genes were identified using information from the literature to analyze their functions. Based on this data, a possible target gene was selected. This selected gene was then queried in the Uniprot database (UniProt Consortium, 2015), and the results were refined by applying a filter for humans (*H. sapiens*). The data was then examined and further analyzed based on family and domain information. As a result, a specific domain was selected as the target protein for further investigation.

### 2.4 Molecular docking

#### 2.4.1 Protein preparation

The target protein SMAD3 (PDB: 1MJS) was downloaded from the protein data bank (PDB) (UniProt Consortium, 2015). CASTp server (Tian et al., 2018) was used to identify the residues, and the CASTp server (Tian et al., 2018) was used associated with binding sites. The protein was prepared using the Autodock tool. The grid box with the following dimensions, 18 Å × 22 Å × 26 Å, was generated to cover the binding site residues. The centre of the grid box was situated at 28.39 Å × 5.22 Å × 4.37 Å along the x, y, and z-axes Hydrogen atoms and Gasteiger charges are added, while water molecules and heteroatoms are removed before docking.

#### 2.4.2 Ligand preparation

The compound library preparation process began with a search of the Selleckchem database for the desired natural substance. (https://www.selleckchem.com/screening/natural-product-library.html). The retrieved compounds were later converted into SMILES for PubChem (Kim et al., 2023) CID using the “PubChem Identifier Exchange Service.” By removing duplicate entries from both conversion processes, distinct CIDs were obtained. Then, the CIDs corresponding to the 3D-SDF structures were acquired using the PubChem API. The other 2D-SDF structures were Collected and converted retrieved and converted to 3D-SDF. After that, the 3D-SDF files were optimized in size using the MMFF94 force field, and Open-babel was used to convert these to PDBQT (O'Boyle et al., 2011). Ligand was converted to PDB from SDF using an open-babel tool. Similarly, open- Babel was used to convert the PDB file to a PDBQT file. The addition of hydrogen was also done by the open-Babel programme.

#### 2.4.3 Ligand-protein docking

The AutoDock 4.2 software (Morris et al., 2009) and AutoDock Vina 1.2.0 (Eberhardt et al., 2021) were used in order to carry out the protein-ligand docking process. AutoDock Vina is a validated and robust molecular docking tool favoured for its advanced scoring function, efficient optimization algorithms, and multithreading capabilities (Trott and Olson, 2010). These features enhance the accuracy and speed of docking simulations, which is crucial for the reliable prediction of binding energies and modes in protein-ligand interactions. The tool’s development focuses on both empirical and knowledge-based potentials, which ensures improved prediction accuracy compared to its predecessors (Forli et al., 2016). Additionally, in the process of conducting docking-based virtual screening, the following characteristics were taken into consideration: twenty binding modes, ten exhaustiveness, and a maximum energy difference of four (kcal/mol).

#### 2.4.4 Molecular dynamics (MD) simulation

The protein-ligand complex has been simulated employing the poses were used for a 100 ns molecular dynamics simulation using Gromacs 2022.4 (2022) (Bauer et al., 2022) (MD). The CHARMM36 force field parameter was used to establish the molecular topology (Huang and MacKerell, 2013). The CGneFF server was used to build topologies and force-field parameters for both the hit molecule and the control inhibitor (Vanommeslaeghe et al., 2010). The Ewald particle mesh method was used to determine the electrostatic force across a specific distance. The system was hydrated using the TIP3P model after being placed into a solvation box (cubic) with a distance of 1.0 nm from the wall (Harrach and Drossel, 2014). The neutralisation process was then carried out using Na^+^ and Cl^−^ ions. After fifty thousand cycles of the steepest descent (SD) method, the system was able to get rid of steric conflicts. Afterwards, the LINCS approach was employed to attain system stability and restrict the bonds (Hess et al., 1997). Furthermore, during a 100 ps simulation period in the NVT ensemble, the system’s temperature was raised to 310 K using a 2 fs timestep. Furthermore, the system was exposed to continuous pressure (NPT ensemble) at 310 K and 1 atmosphere for 1 ns. The simulation was run for 100 ns. The velocity-rescaling technique (Hess et al., 1997) was used to include temperature coupling, while the Parrinello-Rahman pressure method (Hess et al., 1997) was used to provide constant pressure was employed to maintain constant pressure. Hydrogen bonding, root mean square fluctuation (RMSF), and root mean square deviation (RMSD) were evaluated with the use of the GROMACS internal tool during a subsequent post-MD examination.

#### 2.4.5 Principal component analysis (PCA)

Prior to principal component analysis, the trajectory was cleaned by eliminating the periodic boundary condition. For computing the covariance matrix, the Gmx covar tool that is included in the GROMACS package was used. It is possible to utilise the covariance matrix in order to characterise the connection that exists between the atomic variations that are present in the protein-ligand complex. The eigenvalues and eigenvectors of the covariance matrix were obtained via the gmx anaeig function. The following step utilized the GROMACS software, specifically the "gmx anaproj" function, to obtain the PC coordinates for each frame.

### 2.5 Free energy landscape (FEL)

The processes of biomolecule recognition, aggregation, and folding are some of the processes that can be better understood by examining the steady state, which is represented by the minima of the Free Energy Landscape (FEL), and the transient state, which is represented by the barriers of the FEL, in biological systems (Papaleo et al., 2009). The FEL was calculated by calculating the energy distribution according to Eq. 1a:

$$∆G\left(X\right)=-kBTln\;P\left(X\right)$$ (1a)

The variables X, G, kB, T, and P(X) represent the reaction coordinate, Gibbs free energy, Boltzmann constant, absolute temperature, and probability distribution of the system along the reaction coordinate, respectively.

### 2.6 Binding-free energy

When estimating the binding free energy of the protein-ligand complex, the GROMACS add-on tool gmx MM/PBSA was used as the information source (Valdes-Tresanco et al., 2021). The equations applied to compute the MM/GBSA are shown in Eqs 1–6.

$$∆G=G_{complex\;}-\left[\;G_{receptor}+G_{ligand}\;\right]$$ (1b)

$${\Delta G}_{binding}=\Delta H\;–\;T\Delta S$$ (2)

$$\Delta H=\Delta G_{\text{GAS}}+\Delta G_{\text{SOLV}}$$ (3)

$$\Delta G_{\text{GAS}}=\Delta E_{\text{EL}}+\Delta E_{\text{VDWAALS}}$$ (4)

$$\Delta G_{\text{SOLV}}=\Delta E_{\text{GB}}+\Delta E_{\text{SURF}}$$ (5)

$$\Delta E_{\text{SURF}}=\gamma .\text{SASA}$$ (6)

Here, ΔG is defined as the variation in the protein-ligand formation’s Gibbs free energy in Eq. 1b. In the solvent, G_*complex*_ *,* G_*receptor*_ *,* and G_*ligand*_ denote the total free energies of the protein-ligand complex, free enzyme, and ligand, respectively. ΔG_binding_ signifies the modification in the Gibbs free energy corresponding to the protein-ligand binding interaction, while ΔH stands for the modification in enthalpy that incorporates both the gas-phase energy (ΔG_GAS_) and the total solvation free energy (ΔG_SOLV_). The binding free energy, shown as TΔS , is the cumulative sum of the change in entropy. ΔE_EL_ represents a change in electrostatic energy, while $\Delta E_{\text{VDWAALS}}$ signifies the change in van der Waals energy. These two components combine to yield $\Delta G_{\text{GAS}}$ . Moreover, ΔE_GB_ indicates the change in polar solvation energy brought on by polar group interaction, while Δ,E-_SURF_. denotes a change in solvation-free energy brought on by the non-polar interaction. A variation of the solvent-accessible surface area (SASA) was made using the solvent surface tension parameter (γ) in order to compute the solvent-accessible surface area (SURF) of the solvent.

## 3 Results

### 3.1 Healthy vs. active comparison for Duchenne muscular dystrophy (DMD)

**FIGURE 1:**
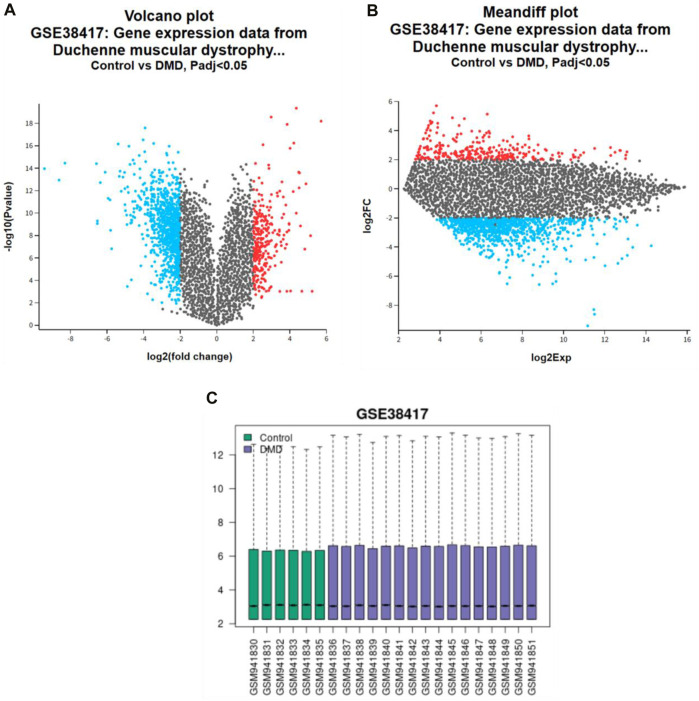
Gene expression analysis in DMD: **(A)** volcano plot, **(B)** MA plots highlighting differential expression between control and DMD cases, with **(C)** Sample distribution bar graphs.

Using microarray datasets from healthy persons and those with active DMD, the current research tried to discover the genes that are expressed differently between the two groups of people. The GSE38417 dataset was derived from the GEO database, which served as its primary source. The GEO2R approach was used to find DEGs in the dataset. A volcano plot is used to show the differential gene expression analysis results (Figure 1A). The x-axis displays the log2 fold change between healthy and DMD conditions; each gene’s expression level varies from condition to condition. The importance of the expression change is shown by negative log10 of the *p*-value, displayed along the y-axis. The genes thought to be more differently expressed are those with greater and further to the left or right points. Following the requirements of an adjusted *p*-value (P*.*adj) less than 0.05 and a logarithmic function (logFC) more than 2, genes that are substantially upregulated are represented by red dots, while genes that are significantly downregulated are represented by blue points. Here, we observed 290 upregulated and 977 downregulated DEGs. Figure 1B) illustrates a mean difference (MA) plot for gene expression data from DMD versus control samples. The y-axis shows the log2FC, log2 Fold change and the x-axis shows the log2Exp, log2 expression. The genes with red dots show a highly differentially expressed gene set, whereas genes with blue points may have modest expression levels but large fold changes.

Figure 1C shows the box plot that compares the distribution of gene expression levels between the healthy (green boxes) and the active DMD (blue boxes) for a series of samples on a logarithmic scale. The centre line in each box shows the median expression level, while the height of the box shows the interquartile range (the middle 50% of data points). There were 1,267 genes found to have differential expression between healthy and active DMD conditions, out of a total of 53,408, with an adjusted *p*-value less than 0.05. These plots were used to analyse and visualise the microarray dataset data and understand the biological significance of gene expression changes in diseases like Duchenne muscular dystrophy. There is a chance that the genes that are elevated (290 DEGs) are responsible for the production of proteins that play an important part in the development of DMD disease. Therefore, the purpose of the research was to examine the dataset GSE38417 in order to identify the DEGs that were elevated.

### 3.2 Protein-protein interaction

**FIGURE 2:**
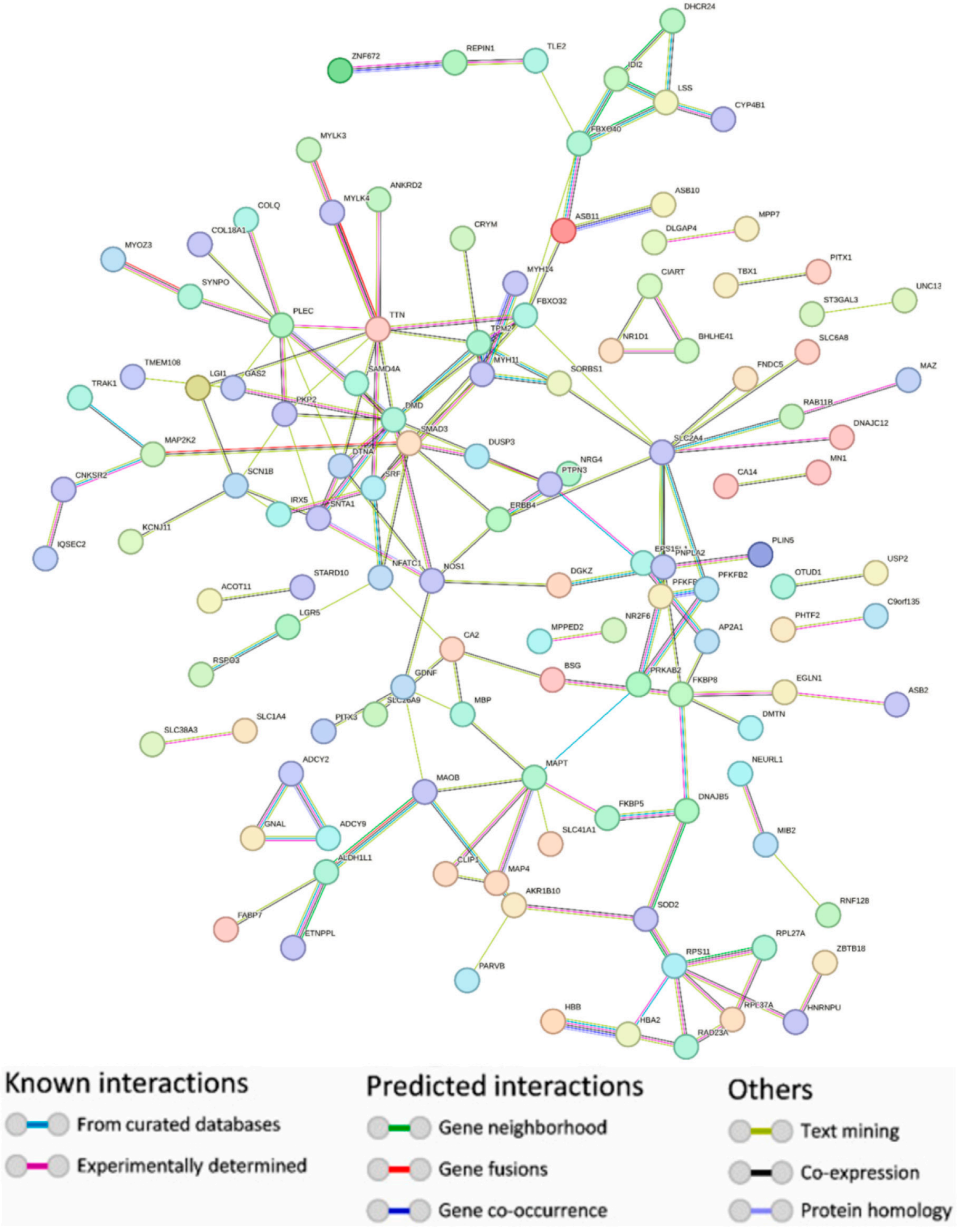
Protein-protein interaction network depicting upregulated genes with hidden disconnected nodes. Node colours indicate different entity classifications, while edge colors represent the nature and source of interactions—ranging from experimentally determined (red) to predicted connections (green) and associations identified through text mining (blue) and co-expression (purple).

**FIGURE 3:**
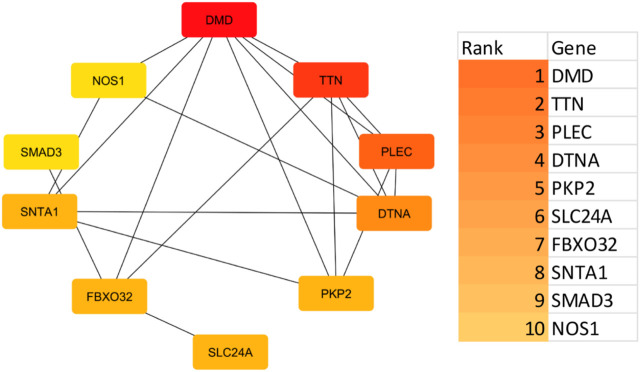
A key regulatory protein interaction network highlights central hub proteins as identified by cytohubba analysis. Gene interaction network with a gradient scale indicating Maximal Clique Centrality (MCC) values for each gene. The gradient bar on the right, ranging from dark orange to light yellow, visually represents the MCC values from highest to lowest, facilitating an understanding of the hierarchical importance of these genes in the network.

Using the STRING platform, the upregulated differentially expressed genes (DEGs) were analysed for protein-protein interactions (PPIs). The medium confidence level was set at 0.90, which is the lowest needed interaction score. Figure 2 illustrates the PPI, which represents the interaction network between DEGs. Disconnected nodes were ignored in the analysis as shown in the Figure 2. Multiple significant nodes and links were among the 290 upregulated DEGs identified from GSE38417. TTN had the highest node degree, with a value of 11. The genes DMD and SLC2A4 exhibited a node degree of 10, which was the second highest among all genes. PLEC and SMAD3 followed this, which was 8 node degree. FBXO32 and MAPT exhibited significant node degree, with a value of 7. The genes were subjected to additional analysis using the Cytohubba plugin of Cytoscape. Cytoscape employed a colour gradient that spanned from red to yellow in order to represent the hubs, thereby signifying their varied degrees of importance within the network. Through this study, the most notable hubs are identified, which are subsequently used in constructing a PPI network. Figure 3 displayed the protein-protein interaction (PPI) network of the top 10 hubs, and Table 1 presented a ranking of the genes based on their scores. Both of these figures were shown in the same document. Figure 3 shows the use of Maximal Clique Centrality (MCC) values to display the gene gradient. The top 10 hubs found by PPI network analysis were DMD, TTN, PLEC, DTNA, PKP2, SLC24A, FBXO32, SNTA1, SMAD3, and NOS1. The DMD gene exhibited the highest MMC score of 29, while the TTN gene scored 23. These two genes, DMD and TTN, are the most significant differentially expressed genes (DEGs) that contribute to the development of muscular dystrophy. The PLEC gene scored 17, the DTNA gene exhibited a score of 12, and the PKP2, SLC24A, FBXO32, and SNTA1 genes exhibited scores of 10. SMAD3 and NOS1 exhibited an MCC score of 9. The top 10 hubs were subsequently used for a literature analysis to examine the association between these genes and muscular dystrophy.

**TABLE 1:** Top 10 hub proteins in network STRING network ranked by MCC method.

Rank	Name	Score	Gene symbol
**1**	ENSP00000354923	29	DMD
**2**	ENSP00000467141	23	TTN
**3**	ENSP00000323856	17	PLEC
**4**	ENSP00000470152	12	DTNA
**5**	ENSP00000070846	10	PKP2
**5**	ENSP00000320935	10	SLC24A
**5**	ENSP00000428205	10	FBXO32
**5**	ENSP00000217381	10	SNTA1
**9**	ENSP00000332973	9	SMAD3
**9**	ENSP00000477999	9	NOS1

### 3.3 Gene ontology

**FIGURE 4:**
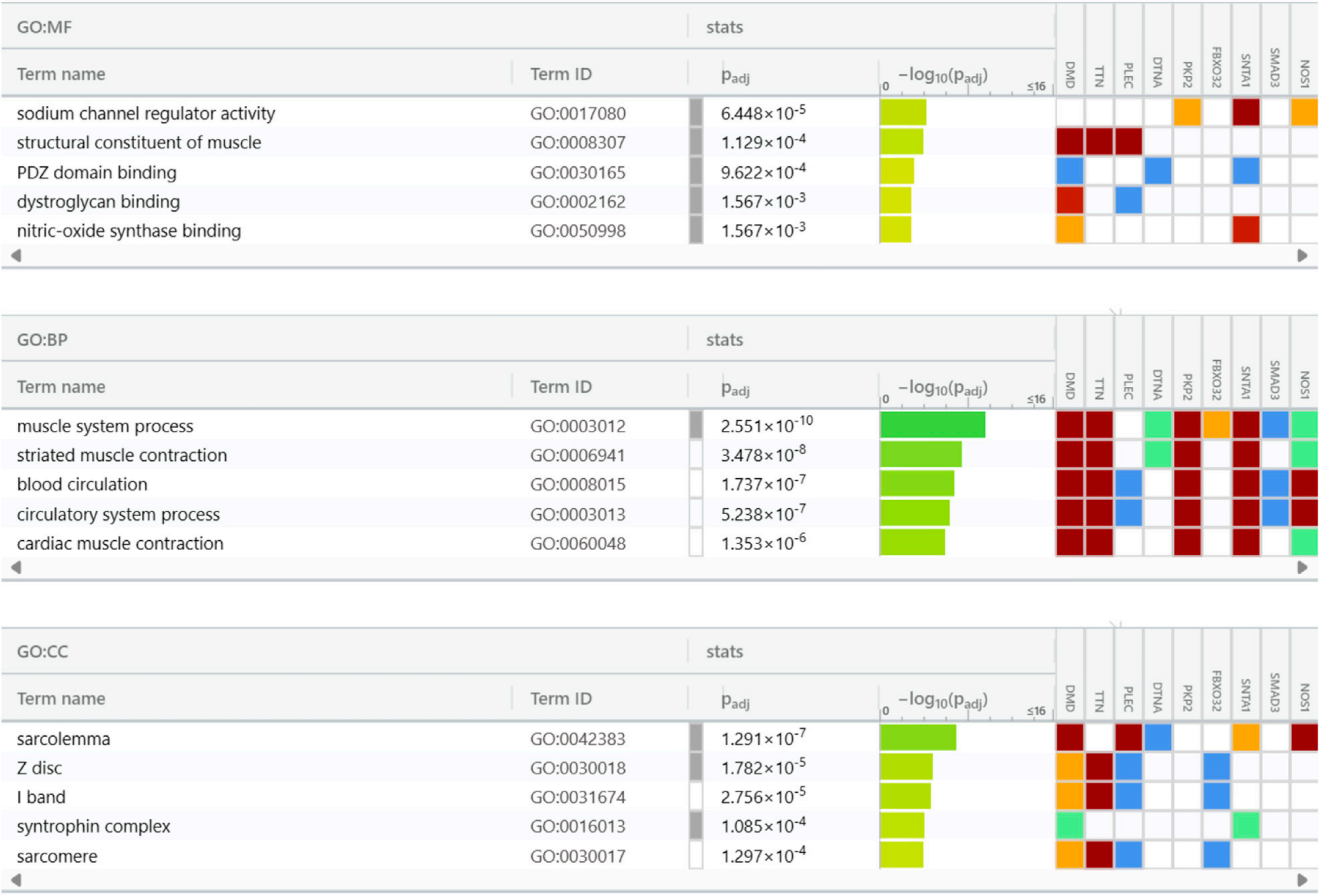
Gene Ontology insights into cellular components (CC), biological processes (BP), and molecular functions (MF) of the top 10 hub genes.

Studies on the enrichment of pathways using Gene Ontology (GO) and the Kyoto Encyclopaedia of Genes and Genomes (KEGG) were carried out with the assistance of the g: Profiler tool. These efforts enhanced the understanding of the biological significance of the upregulated DEGs in the GSE38417 dataset. Examining the functional and route links between these DEGs helped researchers better understand their potential roles in biological systems. The user’s threshold was set to 0.5 in this case, which used the g:SCS threshold. The cellular components (CC), biological processes (BP), and molecular functions (MF) domains were the primary areas of concentration for the GO analysis as shown in Figure 4. Sodium channel regulator activity, PDZ (PSD-95/Dlg/ZO-1) domain binding, dystroglycan binding, nitric-oxide synthase binding, and structural components of muscle were among the molecular processes linked to the DEGs. It was shown that the top ten hub proteins are linked to several biological processes, including those involving the cardiovascular system, striated muscle contraction, blood circulation, and cardiac muscle contraction. The top 10 hub proteins were shown to be connected with several cellular components, including the sarcolemma, Z disc, I band, syntrophin complex, and sarcomere. Duchenne muscular dystrophy (DMD) disrupts a complicated network of interactions and activities, as shown by the association between DEGs and these biological processes and cellular components. Hub proteins have recently been identified, which raises the possibility that they are therapeutic treatment targets due to their potential importance in disease progression. Gaining insight into these connections aids in understanding the underlying pathological processes of DMD and has the potential to identify efficient therapies.

### 3.4 Pathway analysis

**FIGURE 5:**
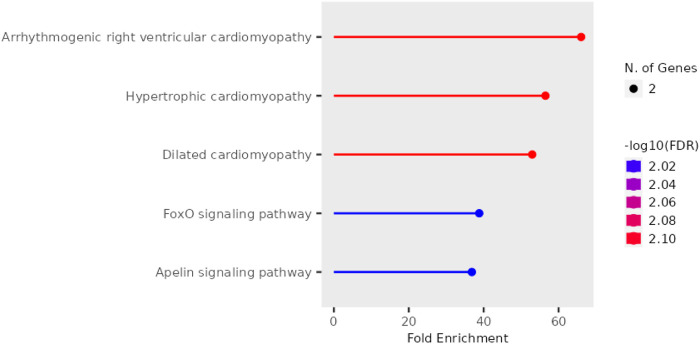
Pathway enrichment analysis of top hub genes identified from the PPI network analysis.

Further, pathway enrichment analysis using the top 10 hub genes was performed. Figure 5 shows that each pathway has a horizontal line ending in a dot, with the dot’s position indicating the fold enrichment. The colour of the dot, which is a statistical metric that is used when testing several hypotheses, correlates to the -log10 of the false discovery rate (FDR). In terms of statistical significance, a deeper shade indicates a greater enrichment than a lighter shade does. The size of the dot is a representation of the number of genes that are involved in the pathway; bigger dots will indicate that there are more genes involved. The arrhythmogenic right ventricular cardiomyopathy and dilated cardiomyopathy pathways exhibit the highest fold enrichment, accompanied by a highly significant FDR (indicated by the red colour). Hypertrophic cardiomyopathy shows a slightly lower fold enrichment but still maintains a significant FDR. On the other hand, the FoxO signaling pathway and Apelin signaling pathway display lower fold enrichments and less significant FDRs (indicated by the blue colour). The enrichment scores and the genes involved in the pathway are listed in Table 2. It was observed that DMD, TTN, and SMAD3 were involved in most of the pathways.

**TABLE 2:** Enrichment scores of key signalling pathways and their related genes.

Enrichment FDR	Fold enrichment	Pathway	Genes
0.007792883	66.03463203	Arrhythmogenic right ventricular cardiomyopathy	DMD, PKP2
0.007792883	56.4962963	Hypertrophic cardiomyopathy	DMD, TTN
0.007792883	52.96527778	Dilated cardiomyopathy	DMD, TTN
0.009610153	38.81424936	FoxO signalling pathway	FBXO32, SMAD3
0.009610153	36.84541063	Apelin signalling pathway	SMAD3, NOS1

### 3.5 SMAD3 a key target in Duchenne muscular dystrophy

The target identified by reviewing various literatures, the upregulation of specific genes in muscular dystrophies, including DMD, might involve intricate interactions between compensatory mechanisms, cellular stress responses, and pathogenic processes. The therapeutic genes involved in DMD were discussed here. The Dystrophin gene is generally not excessively expressed in DMD. However, mutations result in a lack or substantial decrease of dystrophin protein (Duan et al., 2021). Titin (TTN) could potentially be up-regulated as a compensatory mechanism in response to muscle injury. The function of titin in preserving muscle flexibility and structural integrity is critical. In order to preserve the structure and functionality of the muscles, the body may respond to damage to the muscle fibers by up-regulating TTN expression (Loescher et al., 2022). Plectin, or PLEC, serves as a link between the cell membrane and the cytoskeleton, especially in muscle cells. The overexpression of this gene may occur as a reaction to heightened mechanical strain and cellular instability in dystrophic muscles. The overexpression of DTNA may be a way for the dystrophin-associated protein complex to compensate for dystrophin’s loss or malfunction by stabilising the membrane of the muscle cell (Wiche, 1998). The PKP2 gene, also known as Plakophilin 2, has a role in cell adhesion and may be upregulated in muscular dystrophies as a protective reaction to cellular stress and damage, in an effort to preserve cellular integrity (Papaleo et al., 2009). The SLC24A gene family is responsible for encoding sodium/potassium/calcium exchangers. Overexpression may be associated with disrupted ion homeostasis in dystrophic muscles, a prevalent characteristic in muscular diseases.

The FBXO32 gene, also known as Atrogin-1, plays a role in the development of muscular atrophy. Overexpression of this gene in muscular dystrophy may serve as an indicator of continuous muscle loss and atrophy, which are characteristic features of these disorders (Ghasemi et al., 2022). The SNTA1 gene, also known as Syntrophin Alpha 1, is a constituent of the dystrophin complex. Overexpression may arise as a compensatory strategy to stabilise the muscular membrane when functional dystrophin is lacking (Jimenez-Vazquez et al., 2022). NOS1, also known as Nitric Oxide Synthase 1, plays a role in the synthesis of nitric oxide, which serves as a signaling molecule. The overexpression of this gene may be associated with changes in blood circulation or inflammation in muscles affected by dystrophy (Buchwalow et al., 2006). SMAD3 overexpression, which is implicated in TGF-beta signaling, may be associated with fibrosis, a prevalent consequence in muscular dystrophies (Liu et al., 1997). The upregulation of these genes is frequently a reaction to the underlying muscle dysfunction and not a primary instigator of the disease itself. These alterations can be involved in intricate feedback loops where the body endeavors to counterbalance or react to muscle injury, but they can also contribute to the advancement of disease in certain instances. Comprehending these alterations in gene expression is crucial for the development of precise treatments for muscle dystrophies.

SMAD3 overexpression has been associated with muscular dystrophies, according to research. The significance of this discovery lies in the fact that SMAD3 is an essential component of the TGF-β signaling pathway, which plays a role in the processes of tissue remodeling, inflammation, and fibrosis. There are a total of two domains that make up SMAD3. The N-terminus is the location of one of the two, whereas the C-terminus is the location of the other. Because the MH2 domain engages in interactions with a multitude of transcriptional cofactors and the type I TGF-β receptor (TβR-I), it is essential for the activation of transcription by TGF-β. One of the earlier studies indicated that the four particular lysine residues that make up the SMAD3 MH2 domain—Lys333, Lys341, Lys378, and Lys409—were essential to the operation of the domain (Imoto et al., 2005b). Therefore, the goal of targeting the MH2 domain of SMAD3 was to impede the fibrosis process associated with Duchenne muscular dystrophy (DMD).

The SMAD3 gene plays a critical role in DMD, primarily through its involvement in the TGF-β signaling pathway, which is instrumental in processes such as tissue remodeling, inflammation, and fibrosis. These processes are central to the pathology of DMD, where progressive muscle degeneration is compounded by excessive fibrosis and chronic inflammation. The activation of SMAD3 in TGF-β signaling leads to the upregulation of extracellular matrix proteins, contributing significantly to the muscle stiffness and fibrosis seen in DMD patients (Bernasconi et al., 1999; Qin et al., 2011). By modulating SMAD3 activity, there is potential to alter the course of tissue remodeling and inflammatory responses that exacerbate muscle damage (Roberts et al., 2003). Furthermore, the role of SMAD3 in DMD shares mechanistic similarities with its function in cancer metastasis, where it regulates cellular proliferation, migration, and invasion—processes similarly detrimental in DMD albeit through different pathological outcomes (Ismaeel et al., 2019). This crossover highlights SMAD3 as a versatile target in both oncological and muscular pathologies, underpinning its importance across different disease contexts. Targeting the TGF-β/SMAD3 pathway in DMD could therefore mitigate fibrotic tissue development and reduce inflammation, potentially preserving muscle function and improving patient outcomes (Flanders, 2004). Given this pivotal role, targeting SMAD3 presents a promising therapeutic strategy for managing DMD, as evidenced by research models showing reduced fibrosis and improved muscle functions upon modulation of this pathway (Zhou and Lu, 2010). These studies collectively advocate for further exploration of SMAD3 inhibitors as potential therapeutic agents in DMD treatment regimens.

### 3.6 Virtual screening

The MH2 domain of SMAD3 was tested against 2,569 natural compounds. The binding site residues of the MH2 domain of SMAD3 were GLN315, PRO317, ASN320, ALA328, ARG367, THR370, ILE371 and ARG372, as predicted by CASTp server. Top three hits were selected that showed a top binding energy > −9 kcal/mol (Table 3). The three compounds were 12,314,417 (Ojv-VI), 3,874,518 (Hederacoside C), and 5,281,600 (Amentoflavone). These compounds had binding values of −9.6 kcal/mol, −9.5 kcal/mol, and −9.5 kcal/mol, respectively.

**TABLE 3:** Average and top binding scores of the ligands, along with the number of hydrogen bonds (selected compounds are shown in Bold).

Ligands	Average binding energy (kcal/mol)	Top binding energy	Hydrogen bonds
**5,281,600**	−8.43	**−9.6**	**5**
72,950,887	−7.64	−9.6	1
**3,874,518**	−7.97	**−9.5**	**9**
**12,314,417**	−7.84	−**9.5**	**6**
4,485,132	−7.38	−9.4	4
4,463,283	−7.96	−9.3	6
437,080	−7.82	−9.3	6
5,281,847	−8.15	−9.2	3
4,441	−8.08	−9.2	6
328,441	−7.99	−9.2	0

**FIGURE 6:**
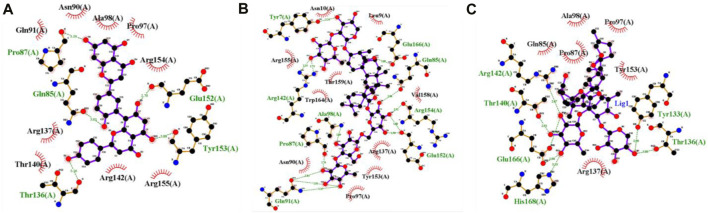
2D interaction representation of the top three docked complex **(A)** 5,281,600 **(B)** 3,874,518 **(C)** 12,314,417 (Green lines with residues indicate hydrogen bonds).

Figure 6 shows the best-docked complexes had protein-ligand interactions. It was observed that 5,281,600 forms five hydrogen bonds with residues Pro87, Gln85, Glu152, Tyr153, and Thr136 (Figure 6A). 3,874,518 forms nine hydrogen bonds with the residues Tyr7, Glu166, Gln85, Arg154, Glu152, Gln91, Pro87, Ala98, and Arg142, (Figure 6B). 12,314,417 forms six hydrogen bonds with the residues Arg142, Thr140, Glu166, His168, Tyr133, and Thr136. The stability of interactions between proteins and ligands is dependent on hydrogen bonds. The identification of the individual residues involved in these interactions can offer valuable insights about the binding mechanism of the compound to the protein and its impact on the protein’s function (Salentin et al., 2014). Therefore, in order to facilitate molecular-dynamic simulation, these three compounds were selected.

The 2D structure of the identified compounds are shown in the Supplementary Figure S1 The Tanimoto similarity of compounds 12,314,417, 3,874,518, and 5,281,600 with SIS3, the known inhibitor of SMAD3 phosphorylation, compared to study the fundamental differences in their mechanisms of action. The values in Supplementary Table S1 suggest that 5,281,600 has low to moderate structural resemblance to SIS3, while 3,874,518 and 12,314,417 show very little similarity. The Tanimoto scores indicate considerable structural differences from SIS3, suggesting that similar biological effects can be achieved through entirely different chemical structures and binding mechanisms.

### 3.7 Molecular dynamics simulation

**FIGURE 7:**
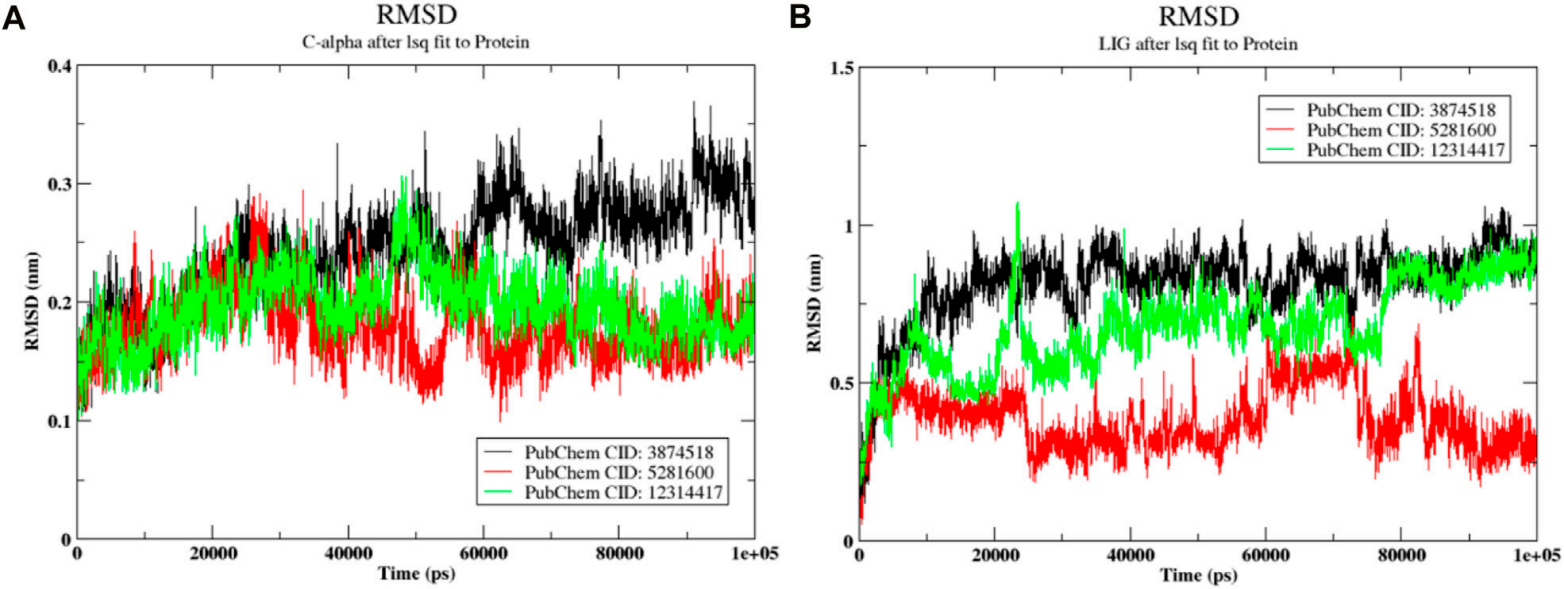
Conformational analysis over the 100 ns molecular dynamics simulation **(A)** RMSD of the Protein (SMAD3) **(B)** RMSD of the ligands.

The post-dynamics simulation study provides critical insights into the flexibility of protein-ligand complexes. During the 100 ns production run, only the best-docked complexes were analyzed. The Root Mean Square Deviation (RMSD) of both the protein and ligand, as shown in Figure 7, highlights the stability and conformational changes over time. These RMSD values indicate how closely the system maintains its initial structure, offering valuable information about the dynamic behavior and potential binding efficacy of the ligand within the active site of the protein.

#### 3.7.1 RMSD

The RMSD is a statistical measure that evaluates the degree toward which protein complexes deviate from their initial structure. This allows for the determination of the overall stability of the complexes. As can be seen in Figure 7A, the magnitude of the protein varied from 0.2 nm to 0.37 nm. The protein in the 3,874,518 complex first showed a variance of between 0.2 and 0.3 nm. After that, the more significant fluctuation rose to a range of 0.32–0.36 nm. With the exception of a single, more notable deviation at 45 ps by 0.3 nm, the protein molecule in the 5,281,600-ligand complex changed consistently between 0.2 nm and 0.25 nm. A stable conformation between 0.25 and 0.24 nm was also observed in the protein combination with 12,314,417. In general, the prediction of a stable conformation in the simulation was supported by the protein RMSD. Figure 7B displays the ligand’s Root Mean Square Deviation (RMSD). At the initial stage, the ligand 3,874,518 exhibited significant fluctuation and reached 0.7 nm, followed by a subsequent period of consistent configuration. Similarly, during the early phase, 12,314,417 exhibited fluctuations with variations reached 0.7 nm and 1.2 nm. On the other side, after 70 ps, it arrived at a stable conformation with a difference of 77 nm regarding the original docked stance. Meanwhile, the 5,281,600 showed deviation ranging from 0.5 nm to 0.4 nm and stability in the last 20 ps. Overall, in the last 20 ps all ligands showed stability.

#### 3.7.2 Conformation analysis

**FIGURE 8:**
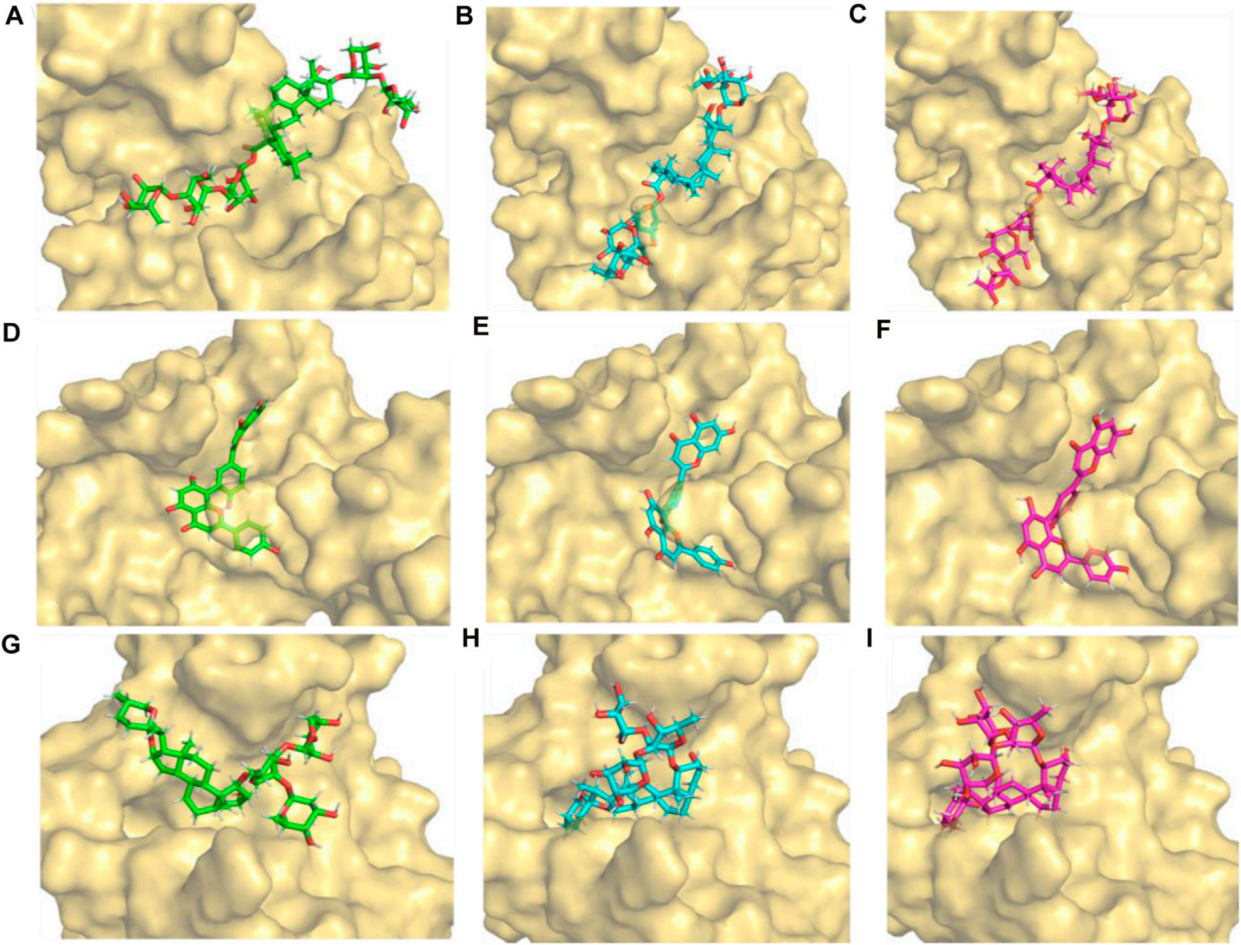
Initial (0 ns), stable (90 ns), and final (100 ns) conformation of the protein-ligand complexes extracted from 100 ns molecular dynamics simulation **(A–C)** 3,874,518 **(D–F)** 5,281,600 and **(G–I)** 12,314,417.

Figures 8A,B,C show the positions of the compounds at 0, 90, and 100 ns during the molecular dynamics simulation, respectively. It was noted that the 3,874,518 showed a deviation from its starting state at 90 ns. However, from 90 to 100 ns, it maintained a similar conformation. Similarly, compounds 5,281,600 and 12,314,417 pose at 0, 90, and 100 ns, as shown in Figures 8D–J, showing a deviation from their starting states at 90 ns. However, from 90 to 100 ns, it maintained a stable conformation.

Despite some early spatial configurational deviations, all three compounds were able to achieve stable conformations by the conclusion of the simulation duration, as seen above. This suggests that, during the course of 100 ns, these compounds’ molecular motions go through an adaptation phase and then enter a stable state.

#### 3.7.3 RMSF and SASA studies

**FIGURE 9:**
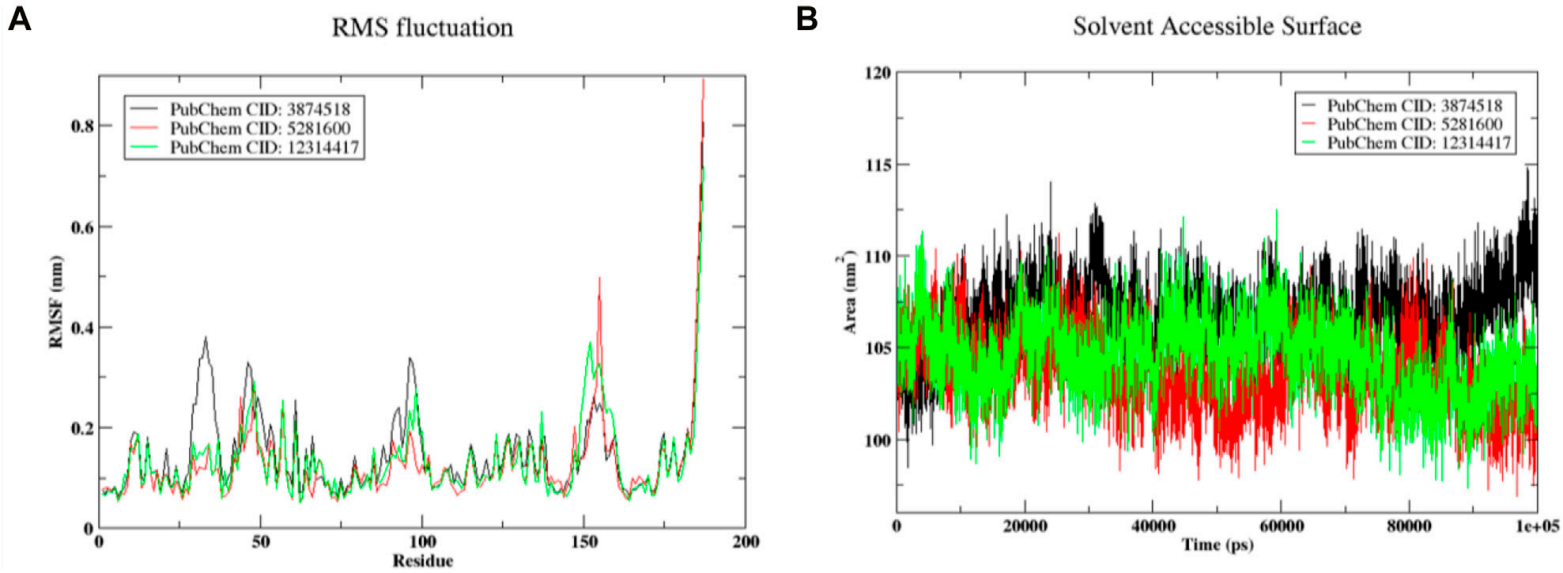
**(A)** RMSF of the complex **(B)** The SASA plot showed change in exposed surface area (on y-axis) with simulation time in picosecond (on x-axis).

Figure 9A displays the protein’s root mean square fluctuation. In this case, it was shown that complexed protein residues with compound 3,874,518 showed larger fluctuations of 0.3 nm than residues with ASP31, PRO32, SER33, ASN34, and SER35. Protein in the complex of compound 5,281,600 showed a higher fluctuation of 0.3 nm, which appears in the residues VAL46 and ASN467. Similarly, protein complexed with compound 12,314,417 showed the maximum fluctuation of 0.3 nm that occurred in the residue HIS96 and PRO97. In general, the complexed form did not exhibit significant changes in protein residues. The SASA plot of SMAD3 is shown in Figure 9B when it is coupled to the ligands 3,874,518, 5,281,600, and 12,314,417. The 3,874,518 complex was shown to have a stable conformation at first, but in the last 20 ns, it diverged, suggesting changes in the solvent-exposed surface area. Similarly, in the initial phase, 5,281,600 showed stable conformation, but at the last 20 ns, it deviated downward from 105 nm^2^ to 110 nm^2^ which indicated the extension of the molecule in the solvent. Additionally, 12,314,417 shows relatively stable SASA, suggesting a stable structure in the solvent during the simulation period. Change in SASA showed the conformational change, however, this change was in the acceptable range.

#### 3.7.4 Hydrogen bonds analysis

**FIGURE 10:**
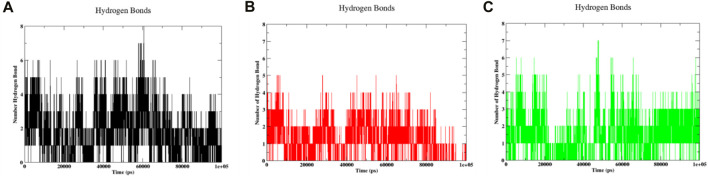
Hydrogen bonds formed during the MD run of 100 ns for **(A)** 3,874,518 **(B)** 5,281,600 **(C)** 12,314,417.

Hydrogen bonds are crucial in molecular dynamics simulations as they significantly impact the structure, function, and interactions of biomolecules within a simulated environment. This facilitates the understanding of their behaviour within actual biological systems (Salo-Ahen et al., 2020). Within the context of the actual case, an estimation of the formation of hydrogen bonds was carried out during the molecular dynamics simulation of the protein-ligand pair. As the ligand 3,874,518 is attached to the protein, Figure 10A shows that it forms 6–8 hydrogen bonds during the 20–60 ns simulation and then progressively reduces to 3–6 hydrogen bonds after 60 ns until 100 ns. Figure 10B depicts the formation of a hydrogen bond when ligand 5,281,600 binds to the protein. It was found that the compound 5,281,600 produced 4–5 bonds within the first 70 ns, however, after that time period, the number of bonds rapidly decreased from 4 to 2. Figure 10C shows the synthesis of hydrogen resulting from the binding of the compound 12,314,417 to the protein. During the initial period of 20 ns, there was a downward trend of sloping down in the formation of hydrogen bonds. However, the value shifted from 20 to 40 ns to 2 to 5. At a time interval of 45 ns, it established a total of 7 hydrogen bonds. The bond formation steadily dropped from 45 ns to of the range of 6 to 4. Throughout the simulation, the amount of hydrogen bonds fluctuated, creating an uneven pattern. However, it is noticeable that detection of H-bonds is highly sensitive to the interatomic distance. These interatomic distances changed marginally during the simulation that can affect the detection of H-bonds. Moreover, the positive side of the observation shown in Figure 10 is that compounds always shown presence of minimum one hydrogen bond.

#### 3.7.5 Principal component analysis (PCA) and free energy landscape (FEL)

**FIGURE 11:**
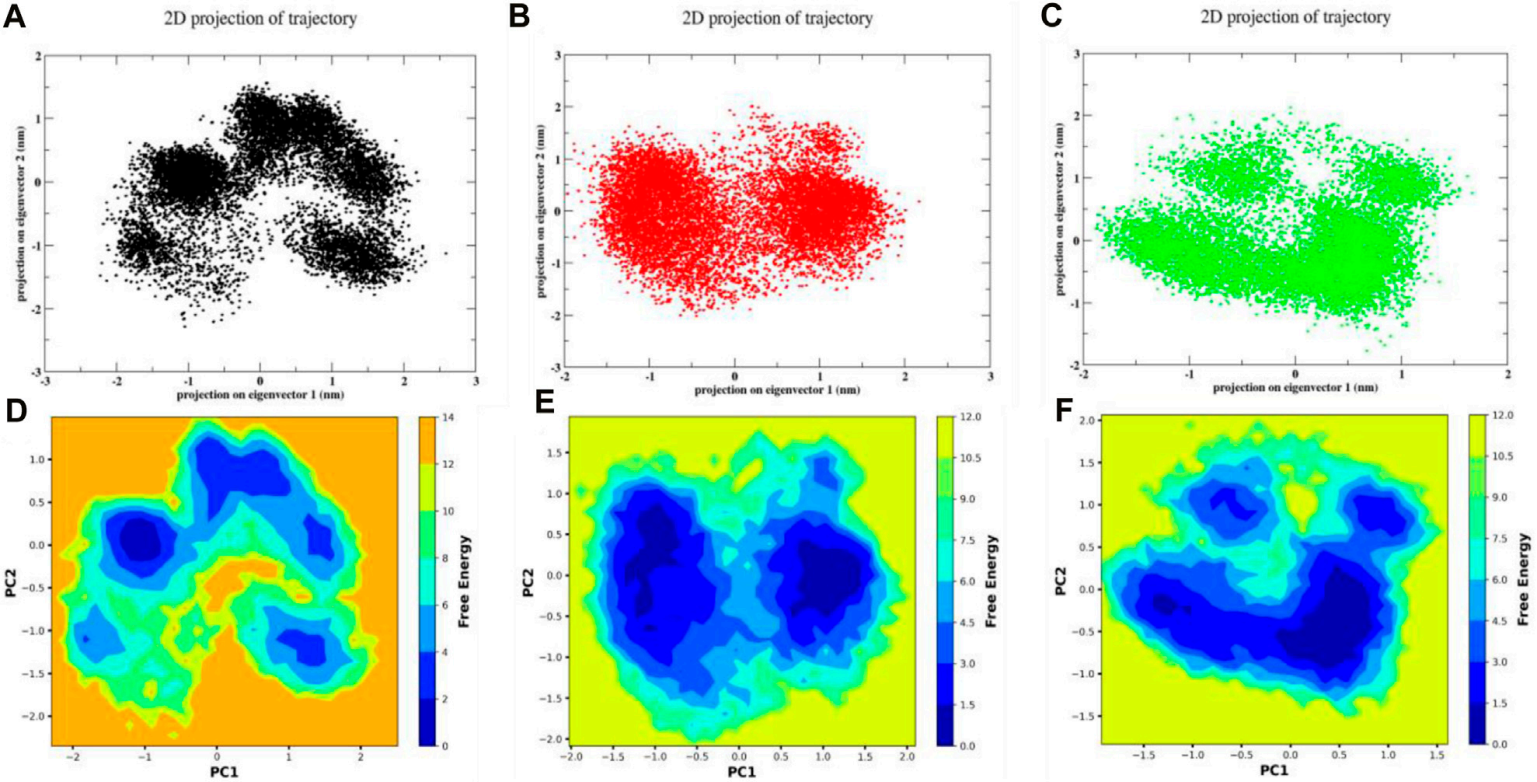
The scatter plot representation of PCA for **(A)** 3,874,518 **(B)** 5,281,600 **(C)** 12,314,417 and FEL of **(D)** 3,874,518 **(E)** 5,281,600 **(F)** 12,314,417.

A scatter plot is used to display the trajectory that was generated by the MD simulation. The Principal Component Analysis is used to provide this plot (PCA). Figure 11A showed the formation of four distinct clusters by molecule 3,874,518 during the MD run, illustrating its transition from its initial to final state. Similarly, the 12,314,417 formed three distinct clusters, but these clusters exhibited a higher level of interconnection as shown inFigure 11C. This suggests that for this compound complex, there was a lower degree of transition compared to the 3,874,518. The 5,281,600 complex formed two closely grouped clusters as shown in Figure 11B, suggesting a higher level of stability compared to other molecules. Additionally, the free energy landscape (FEL) of the 3,874,518, 5,281,600, and 12,314,417, respectively, is shown in Figures 11(D–F). Figures 11(D–F) demonstrates the energy distribution across the conformational landscape. The plot shows regions of low free energy basin in blue, which correspond to more stable conformations or states that the protein-ligand complex frequently occupies. The surrounding areas in orange and yellow represent higher free energy states. The plots represented the stability states of several conformations for the aforementioned system that developed throughout the simulation run. The x-axis showed PC1, while the y-axis plotted PC2. These principal components served as reaction coordinates to show various conformations and their corresponding free energy. Here, in Figure 11C, the plot of 3,874,518 showed four energy basins with multiple energy barriers that suggested an stable conformations of the system separated in the space by barriers. Similarly, Figure 11E 12,314,417 showed three energy basins with multiple energy barriers this suggested unstable system conformation during simulation. However, the FEL of 5,281,600 showed two energy basins with a single energy barrier, as observed in Figure 11D, which indicated stable conformation of the system. Overall, PCA and FEL results indicated that the 5,281,600 could be a strong binder of SMAD3. Overall, complex 5,281,600 and 12,314,417 showed more stable characteristics than 3,874,518 where the conformations are distributed in wider space with few minimas. The total portion occupied by the blue color in the plots were larger for 5,281,600 and 12,314,417 that also suggests larger for 5,281,600 and 12,314,417, suggesting its higher chance of reaching minima.

#### 3.7.6 Binding free energy

**FIGURE 12:**
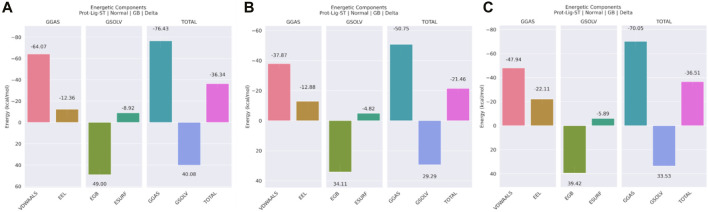
Binding Free Energy using MMGBSA technique representation for the complex of compound **(A)** 3,874,518 **(B)** 5,281,600 **(C)** 12,314,417.

Several different energetic components were used to determine the binding-free energy of the protein-ligand combination. According to the data shown in Figure 12A, the 3,874,518 complex, which is composed of the GGAS and GSOLV, has a total binding energy of −36.34 kcal/mol overall. Moreover, in Figure 12B, total binding free energy was observed as −21.46 kcal/mole for the 5,281,600 complex, and in Figure 12C, total binding energy of the 12,314,417 complex observed as −36.51 kcal/mole. This suggested that the 12,314,417 complex showed strongest binding affinity with the protein molecule. Later, close observation on the different energetic components were made, van der Waals contributed most negatively (−64.07, −37.87, and −47.94 kcal/mol) in all three complexes. This showed that steric clashes were perfectly removed in all the complexes. Electrostatic interaction was also favorable for all the three complexes, in the 12,314,417 complex the electrostatic interaction was best. The complex 3,874,518 and 12,314,417 showed very similar binding energy and emerged as the best ligand to bind with the target protein.

Additionally, the binding free energy of the compounds were calculated using the MM/PBSA technique. Supplementary Figure S2 showed the binding free energy of the complexes. 12,314,417 had the lowest binding free energy was 29.38 kcal/mol 3,874,518 also showed similar binding free energy with 27.81 kcal/mole 5,281,600 had binding free energy of 17.09 kcal/mol. The binding free energy trend was similar in both MM/GBSA and MM/PBSA technique. Morever, binding free energy was calculated for comparative analysis of the three compounds. Among the three compounds, both 3,874,518 and 12,314,417 better binding free energy than 5,281,600.

### 3.8 Discussion

Recent studies on DMD have highlighted gene-targeted medicines, including exon skipping and gene editing, as effective methods to slow down the disease’s advancement. The main goal of these therapies is to repair or substitute the function of the faulty dystrophin gene, which plays a crucial role in DMD pathogenesis (Chung Liang et al., 2022; Moorwood et al., 2011). Advancements in comprehending the interaction between the immune system and skeletal muscles have revealed new possibilities for therapeutic targeting. Studies have emphasised the significance of T-Cell profiling and transcriptome analysis of circulating immune cells in DMD, indicating their potential as new biomarkers or therapeutic targets. This immunological analysis could assist in identifying specific groups of cells that either worsen or can reduce muscle damage, allowing for a more customised therapeutic approach (Molinaro et al., 2024). Research on gene therapy and cell transplantation is ongoing. The procedures entail directly introducing accurate copies of the dystrophin gene or transplanting genetically repaired muscle stem cells into patients. Although these technologies show promise, they also pose notable technological and ethical obstacles such as delivery mechanisms, lasting effectiveness, and possible unintended consequences (Sun et al., 2020).

DMD is characterised by abnormal gene expression, and this work comprehensively evaluates those genes. The gene expression patterns of healthy individuals, as well as those with active DMD, are compared to achieve this. Specifically, the GSE38417 dataset from the Gene Expression Omnibus is used for the study (GEO). In a prior study, Lombardo et al. (2021) and Wu et al. (2022) used a dataset that was quite similar to GSE38417 in order to explore gene modules and their relationships with DMD. Additionally, 290 differentially expressed genes (DEGs) that were upregulated were discovered in the first analysis, demonstrating that substantial gene expression changes connected with DMD. The upregulation genes directly impact the diseased condition. Controlling these upregulated genes would bring down the physiopathology for a given disease, the investigation by Moorwood et al. (2011) revealed that utrophin gene upregulation may be used as a treatment strategy for DMD. Additionally, Kemaladewi et al. (2019) demonstrated that in pre-symptomatic MDC1A mice, up-regulating Lama1 via the CRISPR/dCas9-based approach averted muscle fibrosis and hindlimb paralysis when started early. According to Kemaladewi et al. (2019), it partially reversed the course of the illness and reduced dystrophic features when administered to symptomatic mice with muscle fibrosis and hind limb paralysis that were already evident. The enhanced DEGs were then constructed for PPI networks by using the STRING database and a stringent interaction confidence score threshold of 0.90. This was done in order to guarantee optimal analytical accuracy. It was recognised that the expression of particular genes in DMD might represent a compensatory response to cellular stress or a manifestation of pathogenic processes. Hence, a thorough literature study was used to identify gene targets strategically. The DMD gene typically exhibits low expression in DMD due to mutations that result in a marked reduction of dystrophin protein. Conversely, genes such as TTN, SMAD3 and PLEC may be upregulated in response to muscle injury or cellular instability. However, on the basis of enrichment scores, SMAD3 is identified as the most connected gene. Likewise, Multiple studies have demonstrated that TGFβ1/SMAD3 directly interact with the muscular membrane and inhibits the progression of muscle weakening (Goodman et al., 2013; Zhang et al., 2019). Moreover, Pathway enrichment studies offer a valuable understanding of the functional and pathway connections of these genes that are expressed differently (DEGs), highlighting their involvement in immune-related activities. Consequently, it becomes clear that it might be a great target for the therapy of muscular dystrophy, especially Duchenne muscular dystrophy. It was then tested against a library of naturally occurring chemicals. Natural compound PGC-1 α have shown effective results in DMD conditions by targeting PPARγ (Suntar et al., 2020). Another study demonstrated that Isolecanoric acid (ILA), a natural product, was used to explore its anti-inflammatory effect in DMD conditions (Matias-Valiente et al., 2024). Three compounds in the presented study were identified as the most promising candidates for targeting SMAD3 and its desired domain. The protein molecule in the complex state showed a minimum deviation from its native state, and RMSD was under 0.3 nm during the complete simulation. Studies have shown that under 0.3 nm, RMSD could be considered as minimum and acceptable deviation (Sharma et al., 2021). This confirmed that the binding of the ligand brought some change in the protein conformation, but these were considered stable. However, the RMSD of the ligand molecule was higher than that of the protein molecule. The deviation in the ligand molecule in the protein-bound state is acceptable at the early stage of the simulation, which also underline the limitation of the rigid docking algorithm.

The molecular interactions between molecules 3,874,518, 5,281,600, and 12,314,417, which target the MH2 domain of SMAD3, are essential for influencing the TGF-β signaling pathway implicated in conditions such as DMD, which is distinguished by an excessive accumulation of fibrosis (Chen et al., 2014). These molecules exhibit a strong affinity for the MH2 domain and a low free energy of binding, indicating that they establish stable and energetically advantageous interactions. The stability of these compounds is of the utmost importance for their potential as therapeutic agents, as it indicates that they can consistently and effectively disrupt the normal functioning of SMAD3 within the TGF-β pathway, which is well-known for its substantial contribution to the promotion of fibrosis (Budi et al., 2021). Based on the interaction between these molecules and critical residues in the MH2 domain, it is possible that they could alter the activity of SMAD3 by impeding its normal signaling functions. This could involve impeding essential phosphorylation processes that are required for the activation of genes associated with fibrosis. Consequently, these molecules may reduce the pathological signaling responsible for fibrotic tissue formation, a primary component of DMD pathology, by inhibiting SMAD3. This activity corresponds to the therapeutic objectives of managing and alleviating fibrosis in muscular dystrophies, presenting a potentially fruitful pathway for advancing treatments. Extensive research has been conducted previously on the role of the TGF-β/SMAD signaling pathway in fibrosis across various diseases, underlining the therapeutic potential of modulating this pathway (Bujak and Frangogiannis, 2007; Schiro et al., 2011; Piersma et al., 2015).

The suggested chemical interactions of these drugs with SMAD3 present a potential therapeutic approach for treating disorders marked by excessive fibrosis, such as DMD. Additional empirical research, including thorough biochemical testing and clinical studies, is necessary to validate these results and guarantee the safety and effectiveness of these compounds for human usage. The future applications of targeting the SMAD3 pathway in fibrotic diseases such as DMD are promising and diverse. Precision medicine initiatives could leverage genetic insights to tailor SMAD3 inhibitors to the unique genetic makeup of individual patients, potentially enhancing treatment efficacy and reducing side effects. Insights like these are crucial for creating specific treatments that can control these pathways. The study’s discussion on prospective treatment strategies, like using natural chemicals that target specific genes or pathways critical in DMD, emphasises the study’s translational potential. This component shows promise by indicating potential pathways for translating the findings of this study into clinical therapies. This thorough method improves the comprehension of DMD on a molecular scale and paves the way for future therapeutic advancements focused on more efficient treatment or control of the condition.

## 4 Conclusion

The present study was performed to identify hit molecules targeting the MH2 domain of SMAD3 that is involved in DMD. SMAD3 upregulation showed a significant impact on DMD. Screening of natural compounds found three most promising hit compounds: (a) 3,874,518 (b) 5,281,600 (c) 12,314,417. In the molecular dynamic study, 3,874,518 and 12,314,417 showed stable conformation after initial conformational jump. Combined, these two compounds a protein-ligand complex with a greater binding free energy. The fact that these chemicals remain stable at the protein’s binding site suggests that they may impose some kind of inhibitory mechanism on the protein. Further, experimental assays are required to validate the computational findings.

## Data Availability

The raw data supporting the conclusion of this article will be made available by the authors, without undue reservation.
