# Supplementary material for: Exploring novel natural compound-based therapies for Duchenne muscular dystrophy management: insights from network pharmacology, QSAR modeling, molecular dynamics, and free energy calculations
Source: Front Pharmacol. 2024 Oct 2;15:1395014. doi: 10.3389/fphar.2024.1395014 (PMC11481126; doi:10.3389/fphar.2024.1395014)
Supplement: Supplementary file 1 [file DataSheet1.docx]

**Supplementary**

**Table S1.** Tanimoto similarity of the hit compounds with the known inhibitor of SMAD3, SIS3

| Compounds | AP Tanimoto |
| --- | --- |
| 3874518 | 0.02 |
| 5281600 | 0.22 |
| 12314417 | 0.02 |


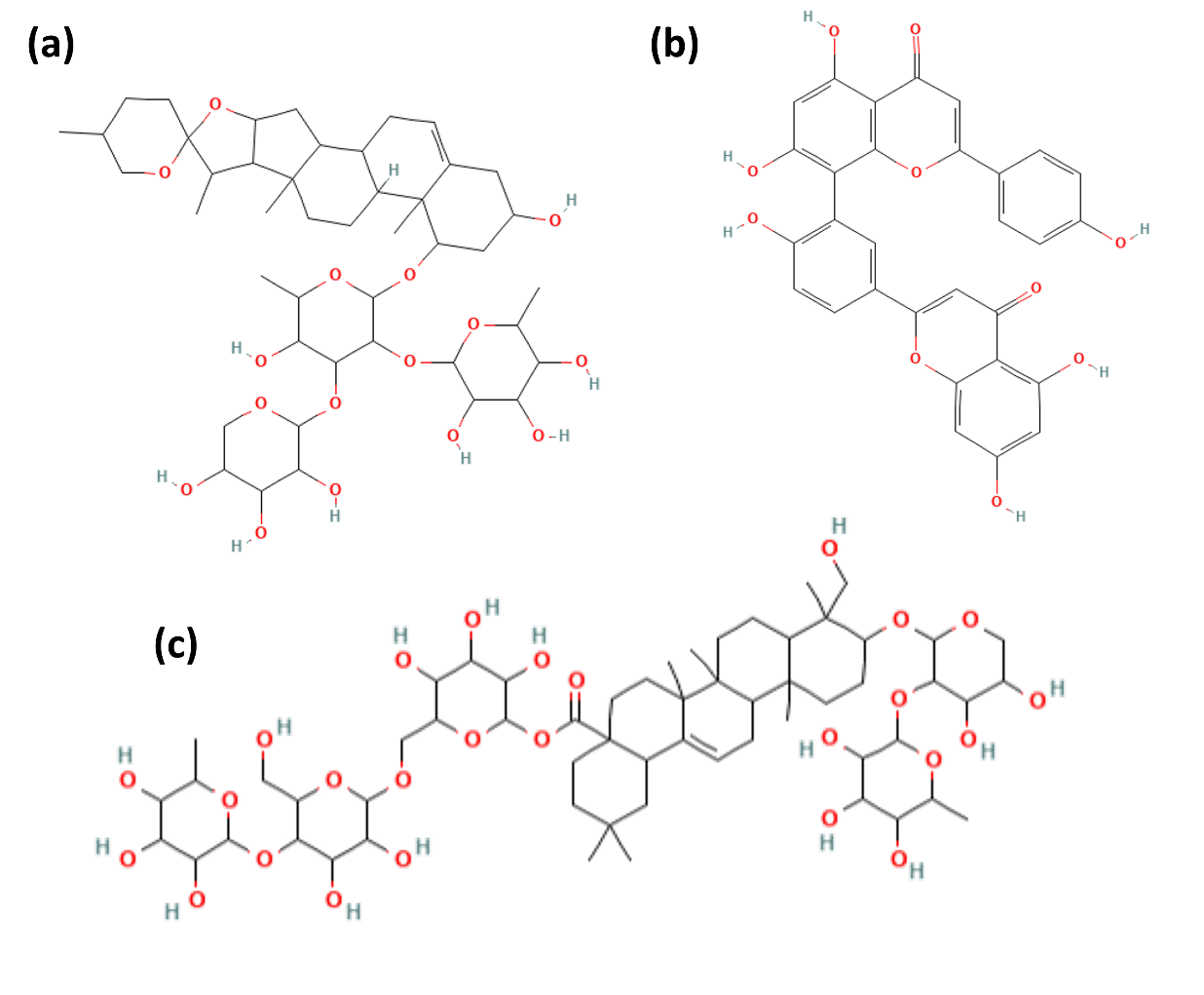


**Figure S1.** 2D representation of the hit compounds (a) 12314417 (b) 5281600 (c) 3874518


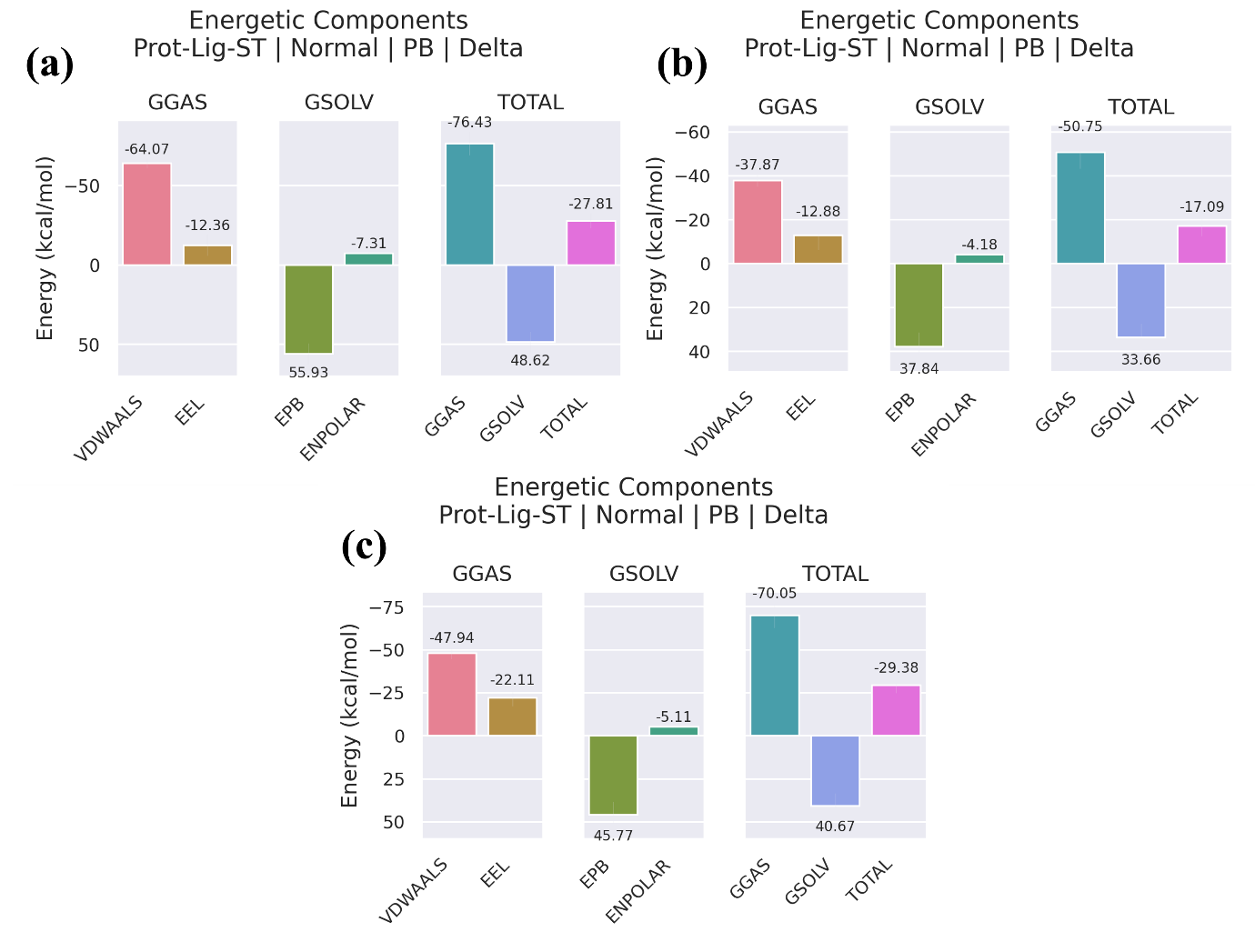


**Figure S2.** Binding Free Energy using MM/PBSA technique representation for the complex of compound (a) 3874518 (b) 5281600 (c) 12314417.
